# Diagnosis of Syphilis by the Microscopic Examination of the Blood

**Published:** 1872-02-15

**Authors:** 


MEDICAL EXAMINER.

*A Semi-Monthly Journal of Medical Sciences.*

EDITED BY

N. S. DAVIS, M. D., AND F. H. DAVIS, M. D.

Chicago, February 15th, 1872.

### EDITORIAL.

### DIAGNOSIS OF SYPHILIS BY THE MICROSCOPIC EXAMINATION OF THE BLOOD.

The Boston *Med. and Surg. Journal*, for Feb. 8th, 1872, contains the following letter from James R. Chadwick, M. D.:

VIENNA, January 12, 1872.

MESSRS. EDITORS:—I have just returned from a meeting of the "Gesellschaft der Aerzte," at which was announced a discovery of the most stupendous importance. I am eager to be the first to herald to the New World through your pages, the greatest success—I may venture to say—as yet achieved by the microscope, to-wit, the certain diagnosis of syphilis. I will give briefly, from memory, the course of procedure which led to this grand result, having first recorded the name of the propounder of the new theory as Dr. Lostorfer. Wishing to make a series of examinations of blood from syphilitic patients, Dr. L. applied to Prof. Zeissl, in August last, who at once placed his wards at the service of the investigator. The manner of conducting the researches consisted in taking a drop of blood, placing it immediately between a slide and covering glass, then setting the specimen under cover. A large number of slides were thus prepared, and daily examined microscopically with a No. 10 immersion lens, Hartnack. The first two days nothing abnormal presented itself; but, on the third, minute shining bodies were discerned, which grew from day to day, until they attained the size of red blood-corpuscles. The shape was, in most instances, round, but modifications of that form were here and there noticed; they were all, however, provided with tapering processes or "sprouts." The number of these corpuscles to be seen at one time within the field of the microscope was variable, amounting in only one specimen to fifty. A month later these objects were still visible. A solution of sugar, as well as several other solutions, caused them to shrivel up, become, as it were, folded upon themselves, lose their shining aspect, and cease to grow. Cold was also found to affect their development, inasmuch as a temperature below 10° Reaumur (54° Fahr.) put a stop to all growth.

To prove the connection between these bodies and syphilis, very many experiments were made with healthy and diseased blood, and the diagnosis confirmed beyond the possibility of doubt.

Blood was then daily examined while the patients were undergoing treatment with mercury (einreibungen), and, after a certain time (in one case twenty-five days), during which the number of corpuscles was steadily decreasing, they had wholly vanished from the field. After these experiments, Dr. Lostorfer submitted himself to Prof. Stricker, and later to Prof. Hebra, to have his power of diagnosticating syphilis, by the microscopic appearances of the blood, put to the test. On various occasions he received from them numbered slides with blood of syphilitic and non-syphilitic individuals. To their astonishment, he unerringly selected from the preparations those where the blood was tainted with syphilis, with the exception that on certain slides the blood was pronounced to be in a condition allowing of no conclusion being arrived at.

The discoverer gives the name of syphilitic corpuscles to these bodies, which, he said, must originate in one of two ways—either from germs always present in the blood, but stimulated to growth by the syphilitic virus alone, or from such introduced bodily into the blood at the time of infection.

In conclusion, he read the records of a number of cases with his microscopic observations of the blood from day to day. As Dr. Lostorfer ceased speaking, a burst of genuine enthusiasm escaped from the assembled physicians, while Prof. Skoda rose to greet the hero of the evening, as the proclaimer of what was destined to prove one of the most important discoveries of the age. He regretted that it was not customary in Austria to give medals or other rewards of merit in such cases, stating that, had this statement been made in Paris, he would have been crowned, as he deserved.

Prof. Stricker detailed the precautions taken to avoid error or deception in each of the trials to which Dr. L. was subjected. The latter's selection of diseased blood was always correct, and decided, except in the few ruined specimens. To ensure the purity of the healthy blood, he had taken his own and that of his servant. After a while, Dr. L. distinguished between these two, inasmuch as the professor's was thicker and "dirtier."

Prof. Hebra confirmed all that had gone before, and said he had been so much struck with the discovery, that Prof. Stricker's blood "contained more filth (schweinerei) than that of any other living man," that he had instituted researches for similar peculiarities among his patients, but, thus far, without result. No words are needed to show the immense field for research, thus suddenly thrown open to investigators, nor can the practical results, likely to proceed from this study, be overestimated. Microscopes all over the world will now be turned toward these new constellations, and their every freak recorded.

Theories of unity and duality will soon cease to vex the earnest student and practitioner, and half the skill and learning of dermatologists is made of no value by this simple and certain indication of the presence of the syphilitic virus. The testimony of two such renowned and incredulous men as Professors Stricker and Hebra is a convincing proof that these corpuscles are not bubbles to be pricked by the next impertinent microscopist. I trust I have not omitted nor misstated any of the points of Dr. Lostorfer's paper, but if so, will send you in expiation of my offence a copy of his article as soon as printed.

Some several years ago a certain Prof. J. H. Saulsbury, of Cleveland, Ohio, announced *a wonderful discovery.* In making some microscopic examinations of syphilitic blood he had found a characteristic living organism, by the presence or absence of which he was able to distinguish, with certainty, the tainted from the healthy blood. In a number of tests applied similar to those described in the above letter, he did seem to make the distinction with ease. He described the objects as round or ovoid bodies with projecting sprouts, etc., very similar to the above. The announcement attracted considerable attention in this country, at least, and a number of our leading microscopists undertook observations with a view of confirming the discovery. Among others, Prof. Freeman J. Bumstead, of New York, had a number of observations made under his direction. Much to the delight of the observers, the wonderful organisms were found to make their appearance in abundance in the blood as soon as it had stood for the requisite time, two or three days; but strange to relate, when the same observations were extended to the interns nurses, assistants, etc., about the hospital the same objects were found to be equally abundant in their blood. Of course, this proved nothing more than the general bad character of hospital employees! Prof. Bumstead, however, we believe was perverse enough to state it as his opinion that these organic growths or sprouts had no connection whatever with syphilis, but were the mere products of decomposition—just as sure to appear in the healthy as the tainted blood.

We record these facts for the enlightenment of our German brethren, and also for the information of the writer of the above letter, who, although an American, seems to be equally unaware of the important fact that we have microscopes and microscopic observers here in this benighted land.

Dr. Lostorfer may, perhaps, have discovered something new—we think more likely, however, that he has merely rediscovered the organisms previously described by Dr. Saulsbury. We shall wait, at any rate, for some further confirmation of the discovery before giving entire credence to it, and, in the meantime, before heralding his own ignorance, with such a grand flourish of trumpets, to the profession throughout the country, would not the author of the above letter have done better had he been sufficiently informed to announce to the German Savans the important fact that this supposed great discovery had probably been already forestalled in his own country?

Alumni Meeting.—The Alumni of the Chicago Medical College will hold their Annual Reunion in the College building, corner of Prairie ave. and 26th street, on Tuesday, March 12th, 1872, at 10 a. m. All Alumni of the College are invited to be present.

Carbolic Acid in Gonorrhœa.—Mr. John Ashmead states that he has been using, for the last five months, carbolic acid in combination with glycerine and tannin, as an injection in cases of gonorrhœa, and has found it quite as efficacious as Mr. Wood described in a former contribution. The formula employed consists of eight grains of carbolic acid, eight grains of tannic acid, half an ounce of glycerine, and water to one ounce. It appears to act as an antiseptic, arrests the discharge, and shortens the course of the disease.

